# How Epigenetics Bridges Genes and Environment: A Multiomics Evidence Chain in Congenital Diaphragmatic Hernia

**DOI:** 10.1002/bdr2.70047

**Published:** 2026-05-13

**Authors:** Yunshan Gao, Xian Zhu, Rutao Dai, Xichen Zhang, Yongyu Ma, Ya Dao, Junru Chen, Shiwu Yang, Jun Wu

**Affiliations:** ^1^ Kunming Children's Hospital Kunming Yunnan China

**Keywords:** congenital diaphragmatic hernia, DNA methylation, epigenetics, gene–environment interactions, miRNA, multiomics integration

## Abstract

**Background/Objectives:**

Congenital diaphragmatic hernia (CDH) is a severe developmental anomaly with high etiological heterogeneity. Although numerous susceptibility genes have been identified, genetic factors alone fail to explain the full disease risk. Epidemiological data highlight the contribution of environmental exposures. Epigenetic regulation provides a crucial link bridging genetic predisposition and environmental influence. This review aims to summarize recent multiomics evidence elucidating how epigenetic mechanisms mediate gene–environment interactions in CDH.

**Methods:**

This narrative review summarizes studies published over the past decade on epigenetic mechanisms in congenital diaphragmatic hernia (CDH), including DNA methylation, histone modifications, noncoding RNAs, and integrative genomic and transcriptomic analyses in human samples, animal models, and iPSC‐derived organoids. Relevant literature was identified through targeted searches of the biomedical literature and screening of reference lists from key articles. Relevant studies were considered according to their relevance to epigenetic regulation, gene–environment interactions, and translational implications in CDH.

**Results:**

Aberrant DNA methylation, histone acetylation imbalance, and dysregulated miRNAs converge on key developmental pathways, including retinoic acid, TGF‐β, and NF‐κB signaling. Experimental evidence shows that miR‐200b supplementation or pharmacological restoration of histone acetylation can partially rescue pulmonary hypoplasia in nitrofen‐induced CDH models. iPSC and organoid systems further demonstrate synergistic effects between genetic susceptibility and mechanical stress, supporting epigenetic regulation as a mechanistic bridge.

**Conclusions:**

Epigenetic mechanisms serve as core mediators linking genetic and environmental factors in CDH pathogenesis. Future directions include large‐scale EWAS/WGBS and multiomics integration, establishment of standardized epigenetic databases, and ethically guided development of prenatal epigenetic diagnostics and targeted interventions.

## 1 Introduction

Congenital diaphragmatic hernia (CDH) is a severe congenital malformation with an estimated incidence of approximately 1 in 2500–3000 live births (Schreiner et al. 2021; Zani et al. 2022). Although advances in perinatal care and neonatal intensive support have improved survival rates for some affected infants, CDH remains associated with significant respiratory morbidity and long‐term complications (Chen, Tao, et al. 2024b; Qiao et al. 2024), imposing a substantial burden on affected families and healthcare systems. Clinically, the marked heterogeneity in CDH presentation suggests that its etiology cannot be attributed solely to a single genetic or environmental factor (Qiao et al. 2021). While previous studies have identified several susceptibility genes (Buczynska et al. 2025; Burns and Kardon 2023; Stokes et al. 2024), collectively, known genetic variants account for only a portion of the overall disease risk (Benincasa et al. 2021). Concurrently, epidemiological evidence links maternal factors—such as nutritional status, medication exposure, and environmental pollutants—to an increased risk of CDH (Aubert et al. 2025; Guan et al. 2022; Sharma et al. 2020); however, the causal mechanisms underlying these associations remain poorly understood (Maia et al. 2022).

Against this background, epigenetics provides a critical entry point for understanding gene–environment interactions (Holden et al. 2025; Petit et al. 2023). Regulatory mechanisms such as DNA methylation, histone modifications, and noncoding RNAs can influence gene expression without altering the DNA sequence (Abdelaziz et al. 2023; Chen, Wang, et al. 2024a) and may mediate the effects of environmental exposures during critical windows of embryonic development (Xie et al. 2021). In recent years, advances in high‐throughput epigenomic sequencing technologies have enabled an increasing number of studies to identify CDH‐specific methylation alterations in placental tissue, umbilical cord blood, and experimental models (Dylong et al. 2023; Jurkowska 2024; Maghin et al. 2022; Meng et al. 2025). These findings suggest that epigenetic modifications may serve as a crucial bridge linking environmental risk factors to developmental anomalies and offer novel clues for potential early diagnosis and intervention (Nolan et al. 2024).

However, current evidence still has several limitations: most studies have small sample sizes (Dai et al. 2024), heterogeneous study populations and tissue sources (Wang et al. 2022), and insufficient reproducibility of findings (Kunisaki et al. 2021); there is a lack of standardized quality control and statistical analysis pipelines across studies (Ullrich et al. 2023); and functional validation remains relatively scarce (Bahado‐Singh et al. 2023). Therefore, a critical narrative synthesis of the existing evidence, together with a summary of common findings, current limitations, and future methodological priorities, is of great significance for advancing the field of epigenetics in CDH.

This narrative review aims to summarize the latest advances over the past decade regarding the role of epigenetics in the pathogenesis and development of CDH, with a focus on how genetic susceptibility and environmental exposures jointly influence disease risk through epigenetic modifications. We critically evaluate the strengths and limitations of the current evidence chain and propose a strategic framework for future research.

## 2 Methods

This review was conducted as a narrative review informed by a structured literature search, rather than as a formal systematic review. Relevant studies on CDH and epigenetic regulation were identified through searches of PubMed/MEDLINE, Web of Science Core Collection, and Scopus, with a primary focus on literature published during the past decade. To provide appropriate biological and historical context, a limited number of earlier landmark studies were also included when directly relevant to key concepts discussed in this review.

The search strategy combined terms related to CDH with terms related to epigenetic regulation and multiomics approaches, including DNA methylation, histone modification, chromatin regulation, noncoding RNAs, gene–environment interactions, organoids, and iPSCs. Reference lists of key articles and relevant reviews were also checked to identify additional relevant studies.

Articles were considered relevant if they addressed epigenetic mechanisms in CDH, developmental pathways with direct relevance to CDH biology, or human, animal, or iPSC‐/organoid‐based evidence relevant to gene–environment interactions or translational implications in CDH. We prioritized recent peer‐reviewed original studies, while selected reviews and methodological papers were included where necessary to contextualize the field and interpret emerging evidence.

Potentially relevant articles were first reviewed by title and abstract, and full texts were consulted when needed to assess their relevance to the scope of this review. Because of heterogeneity in study design, tissue source, model system, and analytical platform across the available literature, the evidence was synthesized qualitatively rather than quantitatively, with emphasis on biological plausibility, mechanistic support, cross‐model consistency, and major methodological limitations.

In keeping with the scope of a narrative review, the aim of this section is to improve transparency regarding literature identification and study selection, rather than to imply formal systematic review methodology.

## 3 Genetic Framework of CDH

CDH, as a complex congenital malformation, has a genetic basis strongly supported by extensive research. A variety of genetic variants have been identified in both syndromic and isolated CDH cases (Aubert et al. 2025) (Table 1). These variants primarily affect key genes involved in diaphragm development and lung morphogenesis, including transcription factors such as GATA4 and ZFPM2, as well as genes in the retinoic acid (RA) signaling pathway (Guan et al. 2022). Notably, loss‐of‐function variants in GATA6 have been shown to disrupt epigenetic regulation during cardiomyocyte differentiation, leading to outflow tract cardiac defects accompanied by diaphragmatic malformations (Sharma et al. 2020). Furthermore, analysis of de novo coding variants in 827 proband‐parent trios revealed a significant enrichment of rare de novo mutations in CDH patients (Qiao et al. 2021). Together, these findings form the foundational genetic framework of CDH.

**TABLE 1:** Congenital diaphragmatic hernia (CDH)‐associated rare and common variants reported in the literature.

Gene/variant	Level of evidence	Related signaling pathway(s)	Key phenotypic associations	References
LONP1	Enrichment of de novo and ultra‐rare inherited variants; candidate risk gene (based on a cohort of 827 CDH patients)	Mitochondrial function (involving peptidase activity); specific signaling pathway not clearly defined	CDH (frequently accompanied by other anomalies, such as cardiac or skeletal defects), high mortality, and requirement for extracorporeal membrane oxygenation (ECMO)	Qiao et al. (2021)
ALYREF	Enrichment of de novo variants; candidate gene (FDR < 0.05)	RNA export and transcriptional regulation; specific pathway not defined	CDH (isolated or complex forms)	Qiao et al. (2021)
MYRF	De novo variants; associated with Cardiac‐Urogenital Syndrome (CUGS)	Retinoic acid (RA) signaling pathway (via modulation of GATA4, WT1, NR2F2, etc.)	Complex CDH (including cardiac and urogenital anomalies), diaphragmatic and pulmonary developmental defects	Qi et al. (2018)
GATA4	Rare variants (familial and sporadic); candidate gene	Transcriptional regulation; potentially involved in heart and diaphragm development	CDH (left‐ or right‐sided)	Yu et al. (2013)
GATA6	De novo mutations; identified by whole‐exome sequencing	Transcriptional regulation; embryonic patterning	CDH (frequently with other anomalies)	Yu et al. (2014)
ZFPM2	Mutations and deletions; high penetrance	Transcriptional regulation (interacts with GATA factors)	CDH (familial or isolated), cardiovascular malformations	Longoni et al. (2015)
PLS3	X‐linked missense variant (gain‐of‐function); validated in mouse models	Actin bundling and cytoskeletal regulation	X‐linked CDH, body wall defects, facial dysmorphism, increased bone density	Petit et al. (2023)
NR2F2 (COUP‐TFII)	Rare heterozygous variants (mostly de novo); validated in mouse models	Transcriptional regulation; embryonic development (e.g., diaphragm formation)	Bochdalek‐type CDH, cardiac defects, developmental delay/intellectual disability, facial dysmorphism, genital	High et al. (2016)
SIN3A	Loss‐of‐function variants; validated in mouse models	Histone acetylation/deacetylation (epigenetic regulation)	Complex CDH, pulmonary hypoplasia, pulmonary hypertension, diaphragmatic developmental defects	Stokes et al. (2024)
HLX, LHX1, HNF1B	CNV associations (patient‐specific or enriched); array CGH studies	Transcriptional regulation; DNA binding and embryonic development	CDH (often with other structural anomalies)	Zhu et al. (2018)
WT1	Conditional knockout mouse models; linked to retinoic acid	Retinoic acid signaling pathway	Bochdalek‐type CDH, diaphragmatic developmental defects	Carmona et al. (2016)
Common variant: SNP rs55705711 (chr3)	Genome‐wide significant in GWAS; included in polygenic risk models	Regulatory regions of developmental genes (e.g., patterning genes)	Increased CDH risk (in European and Latino populations)	Qiao et al. (2024)
Common variant: SNP rs7777647 (chr7)	Genome‐wide significant in GWAS; included in polygenic risk models	Regulatory regions of developmental genes	Increased CDH risk (in European and Latino populations)	Qiao et al. (2024)

Despite substantial progress in identifying CDH‐associated variants, the current genetic framework remains incomplete, highlighting the need to integrate epigenetic and environmental dimensions to explain disease heterogeneity.

## 4 Role of Epigenetic Mechanisms in Development

**FIGURE 1:**
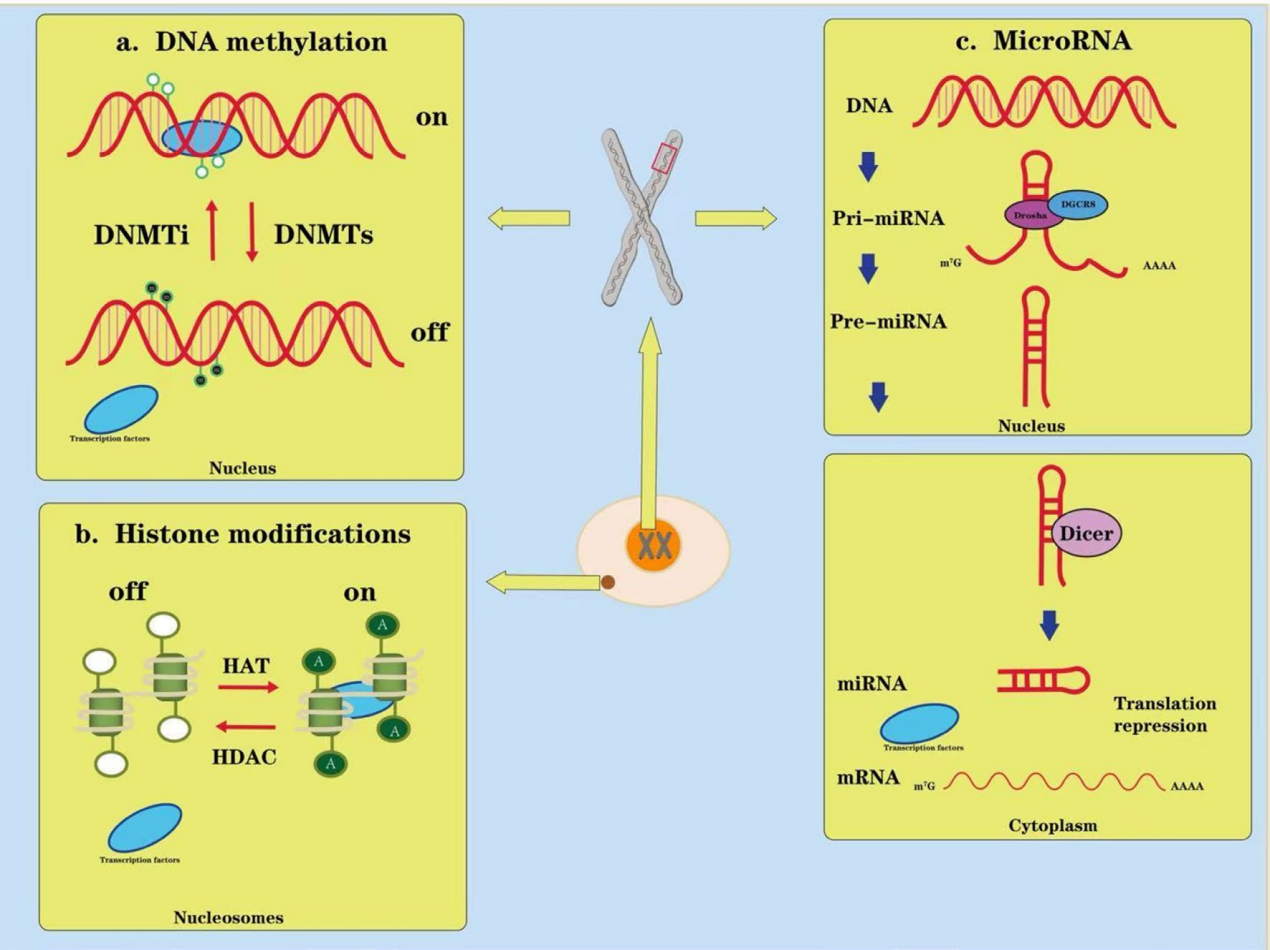
Core epigenetic mechanisms regulating gene expression. Adapted from Xie et al. (2021). (a) *DNA methylation:* DNA methyltransferases (DNMTs) catalyze CpG methylation, which is generally associated with transcriptional repression (“gene off”). Inhibition of DNMT activity (DNMTi) can reduce methylation levels and is associated with transcriptional activation (“gene on”). (b) *Histone modifications:* histone acetyltransferases (HAT) and histone deacetylases (HDAC) dynamically regulate chromatin accessibility through reversible histone acetylation, promoting (“gene on”) or repressing (“gene off”) transcription. (c) *MicroRNA regulation:* primary miRNAs (pri‐miRNAs) are processed by the Drosha–DGCR8 complex to generate precursor miRNAs (pre‐miRNAs), which are further cleaved by Dicer to form mature miRNAs. miRNAs suppress gene expression by binding target mRNAs, leading to translational repression and/or mRNA destabilization. The central schematic nucleus labeled “XX” represents the cellular genome/chromosomal content, illustrating that these epigenetic layers converge on gene regulatory programs during development.

Epigenetic modifications constitute a programmed regulatory system within cells and play a pivotal role during embryonic development. These modifications primarily include DNA methylation, histone modifications (such as methylation and acetylation), and noncoding RNA‐mediated regulation (Chen, Wang, et al. 2024a). Throughout embryogenesis, these epigenetic mechanisms orchestrate complex morphogenetic processes by precisely controlling the spatiotemporal expression patterns of genes. For instance, DNA methylation silences specific developmental genes to maintain cell fate decisions, while histone modifications dynamically regulate chromatin accessibility, enabling the activation of key developmental genes at precise times and locations (Abdelaziz et al. 2023). Noncoding RNAs—such as microRNAs and long noncoding RNAs—further fine‐tune developmental signaling through posttranscriptional regulatory mechanisms (Xie et al. 2021) (Figure 1).

### 4.1 Common Research Approaches

Epigenetic research employs a variety of high‐throughput technologies to comprehensively characterize the epigenome. Epigenome‐wide association studies (EWAS) are a key approach for identifying disease‐associated epigenetic variants, particularly for investigating the relationship between DNA methylation and disease (Jurkowska 2024). Whole‐genome bisulfite sequencing (WGBS) provides single‐base‐resolution maps of DNA methylation across the entire genome, whereas the 450 K/EPIC methylation arrays offer a cost‐effective and high‐throughput solution for large‐scale methylation profiling (Jurkowska 2024). Chromatin immunoprecipitation followed by sequencing (ChIP‐seq) is used to map the genome‐wide distribution of specific histone modifications, while assay for transposase‐accessible chromatin using sequencing (ATAC‐seq) reveals the activity of regulatory elements by assessing chromatin accessibility (Nolan et al. 2024). For noncoding RNA studies, high‐throughput RNA sequencing enables comprehensive profiling of microRNA and long noncoding RNA expression, as well as identification of their target genes (Guan et al. 2022). The integrated application of these technologies provides a multidimensional perspective for elucidating the roles of epigenetic regulation in development and disease (Table 2).

**TABLE 2:** Comparison of epigenetic technologies.

Technology	Main detection object	Resolution	Cost	Batch effect	Sample requirement	Advantages	Limitations/disadvantages
EWAS (chip‐based or sequencing‐based)	Genome‐wide DNA methylation association	Site‐level (depending on platform)	Medium (low for chips, high for sequencing)	Medium, requires strict quality control	Medium–large sample size (hundreds to thousands)	Suitable for large‐scale epidemiological studies, easy to integrate with clinical data	Resolution limited by platform probes; strict control of confounding factors required
WGBS (Whole Genome Bisulfite Sequencing)	Genome‐wide DNA methylation	Single‐base resolution	High	Low (but affected by library preparation)	Large amount of DNA (usually > 1–2 μg), high cost	Highest resolution, covers all CpG sites	High cost, large data volume, complex analysis, not suitable for very large cohorts
450 K/EPIC Array	DNA methylation (specific CpG sites)	~450,000/850,000 CpG probes	Low, cost‐effective	Medium (significant inter‐chip variation)	Medium‐large sample size (100–1000+)	Cost‐controllable, suitable for large‐scale epidemiological studies	Covers only a part of the genome, difficult to discover new regions
ChIP‐seq	Specific histone modifications/transcription factor binding	Peak‐level (hundreds of bp)	Medium‐high	High (many experimental steps, prone to batch effects)	High DNA/cell amount (10^6^–10^7^ cells)	Can reveal the binding map of specific modifications/transcription factors across the genome	Dependent on antibody quality, limited reproducibility and quantification
ATAC‐seq	Chromatin accessibility	Peak‐level (tens to hundreds of bp)	Medium	Medium (library easily affected by conditions)	Low (50,000–50,000 cells, now developed to single‐cell level)	Low sample requirement, suitable for small samples or even single cells	Analysis only provides open regions, does not directly indicate modification types
High‐throughput RNA‐seq (miRNA/lncRNA/mRNA)	Transcriptional products (miRNA, lncRNA, mRNA)	Single‐transcript level	Medium	Medium (library preparation/batch differences significant)	Nanogram‐level RNA	Comprehensive coverage of the transcriptome, including identification of new transcripts	Only indirectly reflects epigenetic regulation, needs integration with other technologies

### 4.2 Analytical Challenges

Epigenetic research faces multiple methodological challenges. Batch effects represent a major source of technical confounding in epigenetic data analysis, particularly when using array‐based platforms; technical variation across experimental batches can obscure true biological signals (Jurkowska 2024). Cellular heterogeneity is another critical issue, as tissue samples typically consist of multiple cell types with distinct epigenetic profiles, necessitating correction through deconvolution algorithms or cell‐type–specific modeling (Nolan et al. 2024). In terms of statistical correction, the problem of false positives arising from multiple hypothesis testing requires stringent control, commonly addressed using methods such as the false discovery rate (FDR) (Jurkowska 2024). Furthermore, the dynamic nature and tissue specificity of epigenetic marks add substantial complexity to data interpretation and integration (Dai et al. 2024). In developmental studies, analyzing epigenetic changes across time‐series samples introduces additional challenges related to modeling temporal dependencies and developmental trajectories (Wang et al. 2022). Addressing these methodological issues is essential for generating robust and biologically meaningful findings in epigenetic research.

## 5 Epigenetic Evidence in CDH

Accumulating epigenetic evidence from diverse sample sources indicates that epigenetic modifications play a significant role in the pathogenesis and phenotypic heterogeneity of CDH, although the strength of evidence varies considerably across different lines of investigation. First, clinical sample studies have shown that circulating microRNAs (miRNAs) are associated with pulmonary hypertension and chronic lung disease in CDH patients—for example, as reported by Herrera‐Rivero et al. These human association studies suggest that miRNAs may serve as potential biomarkers. However, most are based on small sample sizes and lack validation in independent cohorts, making it difficult to draw causal conclusions based on this evidence alone (Herrera‐Rivero et al. 2018).

Second, strong preclinical functional evidence comes from intervention studies targeting miRNAs. Multiple animal experiments have demonstrated that prenatal administration of miR‐200b—either directly or via in vivo delivery systems—suppresses the TGF‐β signaling pathway and partially rescues lung developmental defects induced by nitrofen, indicating that miRNAs are not merely correlative biomarkers but also possess actionable therapeutic potential (Khoshgoo et al. 2019). Recent in utero delivery studies have further shown that miRNA administration can simultaneously induce epigenetic modifications and ameliorate pulmonary phenotypes, thereby establishing a causal evidence chain linking “epigenetic alteration → functional recovery” (Ullrich et al. 2023).

Third, mechanistic studies focusing on chromatin and histone modifications have provided direct evidence linking genetic variants to epigenetic states and ultimately to phenotypic outcomes. Taking SIN3A as an example, both human sequencing variants in CDH patients and tissue‐specific knockout in mice demonstrate that SIN3A is essential for maintaining histone acetylation homeostasis during lung and diaphragm development. Critically, treatment of SIN3A‐mutant mice with a histone acetyltransferase (HAT) inhibitor—specifically, anacardic acid—partially restored lung development and alleviated pulmonary hypertension, thereby establishing a complete evidence chain from “gene → epigenetic dysregulation → pharmacological rescue” (Stokes et al. 2024). Such studies offer direct mechanistic support for targeting epigenetic regulation as a therapeutic strategy in CDH.

Moreover, hyperactivation of inflammation‐related pathways—particularly NF‐κB—has been consistently demonstrated in both human CDH lung tissues and animal models, as well as in ex vivo lung explant experiments (Dylong et al. 2023). Pharmacological inhibition of this pathway, using agents such as dexamethasone or specific NF‐κB inhibitors, has been shown to restore branching morphogenesis in lung explants, suggesting that the crosstalk between inflammation and epigenetic regulation represents a tractable, druggable axis (Stokes et al. 2024).

Collectively, miRNA‐based interventions, restoration of histone acetylation balance, and suppression of inflammatory signaling currently constitute the three most robust translational avenues supported by functional evidence. However, each of these lines of inquiry still lacks validation in large‐scale human cohorts, representing a critical gap in moving these findings toward clinical application (Table 3).

**TABLE 3:** Epigenetic evidence in CDH.

No.	Evidence (pathway/biomarker)	Key study	Sample/model (*n*)	Key methodology	Main findings (key points)	Functional validation/replication	Limitations	Assessed evidence strength
1	Circulating/local microRNAs (clinical samples: neonatal blood, tracheal aspirate, amniotic fluid)	Herrera‐Rivero et al. (2018)	Human: direct pulmonary vein/peripheral blood/neonatal samples (small cohort, ~18 cases)	miRNA sequencing/expression profiling (qPCR validation)	Specific circulating miRNAs associated with CDH and correlated with pulmonary hypertension and chronic lung disease; suggest potential as prognostic/phenotypic biomarkers	Statistical association + small‐scale validation (no independent large‐cohort replication)	Small sample size; correlational only (lacks causality); confounded by tissue heterogeneity	Moderate—clear clinical relevance but requires large‐scale replication
2	miR‐200b (therapeutic intervention/functional validation in animals)	Khoshgoo et al. (2019)	Nitrofen‐induced rat/rabbit CDH models (multiple in vivo/in vitro experiments)	miRNA mimics delivery (transplacental/in utero), lung explants, pathway analysis	miR‐200b upregulation suppresses TGF‐β/SMAD signaling and improves lung development, reducing CDH incidence or mitigating pulmonary hypoplasia (consistent in vivo and in vitro)	Clear functional rescue (consistent across models); reversible therapeutic effect demonstrated	Animal models only—caution needed for human translation; dosing, safety, and delivery routes require further evaluation	Strong—robust preclinical evidence with mechanistic and therapeutic support
3	Extracellular vesicle (EV)‐associated miRNAs in amniotic fluid/tracheal aspirate (prenatal biomarkers)	Fabietti et al. (2021)	Human: severe CDH fetuses undergoing FETO (limited sample size)	Nanoparticle tracking analysis (NTA), OpenArray miRNA screening, qPCR validation	Higher EV counts and specific EV‐encapsulated miRNAs (e.g., miR‐223‐3p, miR‐503‐5p) associated with poor outcomes; suggest EV‐miRNAs as severity/prognosis markers	Technical validation + correlation with clinical outcomes (single cohort)	Rare samples; limited to FETO cases; biological origin and mechanisms not fully elucidated	Moderate—direct human evidence with biological plausibility, but needs multicenter validation
4	Histone acetylation/chromatin regulation (SIN3A → HAT balance): gene–epigenetic interaction	Stokes et al. (2024)	Conditional *Sin3a* knockout mice + pharmacological intervention (anacardic acid)	Transgenic models, histology, molecular markers, HAT inhibitor rescue	*Sin3a* loss disrupts lung/diaphragm development and histone acetylation homeostasis; HAT inhibition partially rescues lung morphology and pulmonary hypertension	Clear gene → epigenetic → phenotype → pharmacological rescue chain (in vivo)	Mouse model—human relevance requires confirmation; long‐term/transgenerational effects unassessed	Strong—complete mechanistic chain with druggable epigenetic target
5	Inflammatory transcription factor NF‐κB hyperactivation (epigenetic–inflammation crosstalk)	Dylong et al. (2023)	Nitrofen rat model + human fetal lung explants	Immunohistochemistry, phospho‐NF‐κB detection, lung explant branching assays, drug intervention (dexamethasone/curcumenol)	NF‐κB overactivation in CDH lung epithelium (human and animal); NF‐κB inhibition restores branching morphogenesis and molecular markers	Consistent findings in human tissue and animal models; pharmacological reversibility (ex vivo/in vivo)	Inflammatory network complexity; direct epigenetic marks (methylation/histone) linked to NF‐κB not fully mapped	Moderate to strong—functional evidence for intervenable epigenetic–inflammation axis
6	iPSC‐derived lung organoids (LO): gene–mechanical–epigenetic interplay	Kunisaki et al. (2021)	Human iPSCs from Bochdalek CDH patients/fetuses → LO (multiple clones, *n*≈10 experiments)	iPSC differentiation, LO modeling, mechanical compression, transcriptomic profiling	CDH‐derived LOs show reduced NKX2.1^+^ progenitors, AT2, and PDGFRα^+^ cells; mechanical stress alters gene expression—supports “intrinsic susceptibility + extrinsic mechanical insult” model	Human‐derived model + in vitro mechanical simulation; platform for gene–epigenetic studies	Primarily transcriptomic; limited direct epigenomic (methylation/histone) data	Moderate—high‐fidelity human model supporting transcriptional/epigenetic dysregulation; needs deeper epigenome profiling
7	Genetic variants in CDH implicate epigenetic regulators (e.g., *EP300*, *SIN3A*)	Scott et al. (2022)	Large trio cohorts/meta‐analyses	WES/WGS, gene enrichment, functional annotation	De novo/rare variants enriched in chromatin/epigenetic regulators; genes like *LONP1*, *ALYREF* implicated; epigenetic factors recurrently flagged in CDH gene sets	Genetic evidence suggests involvement; most variants lack functional validation	Gap between genetic hits and direct epigenetic readouts; multiomics integration needed	Moderate—strong genetic signal pointing to epigenetic machinery, but mechanistic bridge incomplete
8	(Gap) Large‐scale human EWAS/WGBS specifically for CDH	— (no major published studies to date)	—	—	No robust, reproducible large‐cohort EWAS or WGBS studies focused on CDH (e.g., placenta, cord blood) have been reported; only indirect or small methylation studies exist	—	Critical evidence gap—large, standardized multicenter EWAS/WGBS urgently needed	Limited/absent—represents a major weakness in current epigenetic evidence for CDH

## 6 Epigenetics as a Mediator of Gene–Environment Interactions

### 6.1 Epidemiological Evidence

Epidemiological studies have consistently identified multiple maternal exposures associated with an increased risk of CDH. Among these, maternal vitamin A deficiency and dysregulation of its active metabolite, RA, show the strongest and most biologically plausible association with CDH (Beurskens et al. 2013; Michikawa et al. 2019). Animal models have demonstrated that disrupted RA signaling leads to defective diaphragm development, underscoring the pathway's essential role in embryogenesis (Burns and Kardon 2023; Clugston et al. 2010; Montedonico et al. 2006).

Tobacco exposure—whether before conception or during any stage of pregnancy—has also been robustly linked to elevated CDH risk, with reported odds ratios (OR) ranging from 1.5 to 2.0, and evidence of a dose–response relationship (Yang et al. 2022). While direct evidence linking tobacco‐induced epigenetic alterations to CDH is still emerging, maternal smoking during pregnancy has been repeatedly associated with durable epigenetic changes (e.g., differential DNA methylation) in the offspring, providing a plausible mechanistic framework for developmental toxicity (Rogers 2019). This suggests a direct teratogenic effect of smoking‐related compounds on fetal development.

Furthermore, maternal asthma (MA) has been shown in respiratory disease research to alter fetal developmental programming through epigenetic mechanisms, such as DNA methylation changes in immune and lung development genes (Magnaye et al. 2022). Given the shared developmental pathways between lung and diaphragm formation, similar epigenetically mediated mechanisms may contribute to CDH pathogenesis.

Notably, environmental pollutants—including nitrogen oxides—have been shown in experimental models to induce CDH‐like phenotypes (Aubert et al. 2025). Clinically, maternal metabolic conditions such as obesity are increasingly associated with CDH, and emerging evidence suggests this link may be mediated through epigenetic reprogramming of metabolic and developmental genes in the fetus (Buczynska et al. 2025). Together, these epidemiological findings support a model in which environmental exposures interact with genetic susceptibility via epigenetic modifications to influence CDH risk.

### 6.2 How Epigenetic Evidence Links Environmental Exposures to Genes

Epigenetic mechanisms provide a testable mediating model: maternal or prenatal environmental exposures (nutritional deficiency, smoking, pollution, or mechanical compression) → alterations in epigenetic marks in the embryo or placenta (DNA methylation, histone modifications, ncRNA expression) → spatiotemporal misexpression of key developmental genes → ultimately resulting in anatomical and functional abnormalities of the diaphragm and lungs. Multiomics and model system studies have provided partial support for this cascade: on one hand, human‐derived liquid biomarkers (miRNAs in EVs, amniotic fluid, or amniotic membrane fluid) reflect fetal stress states and correlate with clinical outcomes (Fabietti et al. 2021); on the other hand, iPSC‐derived lung organoids (LOs) show that genetic susceptibility and mechanical compression synergistically influence cell lineage decisions, suggesting that environmental stress can amplify genetic predisposition at both transcriptional and translational levels (Kunisaki et al. 2021).

Specific mechanistic studies have shown that maternal vitamin A deficiency leads to aberrant expression of key diaphragm developmental genes such as GATA4 and ZFPM2 by reducing DNA methylation levels in their promoter regions (Sharma et al. 2020). In smoking exposure models, genome‐wide methylation analyses revealed altered methylation patterns of TGF‐β pathway–related genes (e.g., miR‐200b) in lung tissues of CDH fetuses, thereby impairing alveolar branching morphogenesis (Zhu et al. 2025). Mechanical stress studies further demonstrated that impaired generation of NKX2.1 progenitor cells in LOs derived from CDH fetuses is associated with aberrant histone modifications, which disrupt PDGFRα+ myofibroblast differentiation (Kunisaki et al. 2021). Notably, abnormal co‐localization of PRC2‐mediated H3K27me3 and H3K9me was identified in diaphragm tissues from CDH patients, suggesting that this unique chromatin state may account for the incomplete penetrance observed in some individuals carrying pathogenic genetic variants (Giuffrida et al. 2023).

In causal inference, observational EWAS alone are insufficient to establish causal pathways; thus, genetic instruments—such as methylation quantitative trait loci (meQTLs) or GWAS‐derived instrumental variables—must be integrated with mediation analysis or Mendelian randomization to mitigate confounding and reverse causation. Concurrently, functional validation—such as locus‐specific epigenome editing using dCas9‐TET/DNMT systems or cross‐species verification in iPSCs, organoids, and animal models—is a critical step in elevating associative signals to actionable therapeutic targets. Indeed, several functional intervention studies targeting miRNAs and histone‐modifying factors have already demonstrated a feasible path from association to causal and therapeutic validation (Stokes et al. 2024; Ullrich et al. 2023).

## 7 Functional Validation and Research Models

### 7.1 Animal Models

Animal models play a pivotal role in the study of CDH, particularly vitamin A (RA) deficiency models and pharmacological exposure models. Evidence indicates that maternal deficiency of vitamin A and its derivative, embryonic RA, is closely associated with CDH pathogenesis (Petit et al. 2023). In mouse models, tissue‐specific knockout of epigenetic regulatory genes results in key CDH features, including diaphragmatic defects, pulmonary hypoplasia, and pulmonary hypertension (Stokes et al. 2024). Moreover, disrupted RA signaling profoundly alters the expression of genes critical for diaphragm development (Khalaj et al. 2022). The nitrofen‐induced rat model has also been widely used to investigate CDH mechanisms, revealing specific cellular phenotypes and defects in developmental signaling pathways during lung morphogenesis (Aubert et al. 2025; Dedeloudi et al. 2025). These animal models not only recapitulate the pathological hallmarks of human CDH but also provide essential platforms for exploring gene–environment interactions.

### 7.2 iPSCs and Organoids

Induced pluripotent stem cells (iPSCs) and organoid technologies offer new opportunities for studying CDH. Studies using LOs derived from fetuses and infants with Bochdalek‐type CDH have demonstrated that these models can recapitulate disease‐associated cellular deficits, including impaired generation of NKX2.1^+^ progenitor cells, alveolar epithelial type II cells, and PDGFRα^+^ myofibroblasts (Kunisaki et al. 2021). These organoid models can also mimic disease‐relevant mechanical stress, providing a unique platform to investigate how environmental factors influence gene expression (Kunisaki et al. 2021). Single‐cell RNA sequencing has been applied to fetal CDH lung tissue in rat models, revealing differential responses to therapy across distinct pulmonary cell compartments (Antounians et al. 2024). These innovative models hold promise for bridging the critical gap between genetic discovery and functional validation.

Despite notable progress, the CDH research field continues to face significant challenges in functional validation. A standardized pipeline bridging genomic discovery to functional confirmation is currently lacking (De Bie et al. 2022). Most reported CDH‐associated genetic variants have not been subjected to thorough mechanistic investigation (Aubert et al. 2025; Qiao et al. 2024). Establishing a multitiered validation framework—integrating iPSCs, organoids, and animal models—is essential for elucidating CDH pathogenesis (Stokes et al. 2024). Moreover, inconsistencies in animal models and experimental protocols across laboratories hinder direct comparison of findings (Aubert et al. 2025; Dedeloudi et al. 2025). Future efforts must focus on developing standardized functional validation workflows, including unified phenotypic assessment criteria, reproducible experimental conditions, and cross‐model validation strategies, to accelerate both mechanistic understanding and therapeutic development for CDH.

## 8 Challenges and Methodological Considerations

### 8.1 Sample Scarcity and Limited Statistical Power

CDH is a relatively rare but clinically severe congenital malformation and presents a major challenge in epigenetic research due to the difficulty in obtaining sufficient human samples. Current studies largely rely on fetal tissues obtained during surgical repair or samples from animal models (Aubert et al. 2025; Kunisaki et al. 2021), which are limited in quantity and often exhibit pronounced batch effects. For instance, even in a large exome sequencing study of 827 proband‐parent trios, only a small number of disease‐associated genes reached statistically significant association (Dylong et al. 2023), highlighting the issue of limited statistical power. Furthermore, CDH patients frequently present with additional structural anomalies or neurobehavioral features (Antounians et al. 2024), and this clinical heterogeneity adds further complexity to study design. To date, the majority of CDH cases still lack a definitive genetic diagnosis (Dedeloudi et al. 2025), underscoring the need for large‐scale, multicenter collaborations to enhance statistical power and accelerate discovery.

### 8.2 Multiomics Integration and the Need for Cross‐Cohort Standardization

Investigating the role of epigenetic mechanisms in CDH requires integrated analysis of genomic, epigenomic, and transcriptomic data. Existing studies have identified sex‐specific regulatory patterns of the histone modification mark H3K27ac during lung development (Chater‐Diehl et al. 2021) and demonstrated the critical role of microRNA‐200b in modulating lung branching morphogenesis via the TGF‐β pathway (Sugar et al. 2021). However, multiomics integration faces significant challenges due to inconsistent analytical approaches: a benchmarking study evaluating 12 integration methods revealed substantial differences across algorithms in both visualization quality and quantitative performance metrics (Suraweera et al. 2025). In CDH‐specific research, the nitrofen rat model and surgical lamb model have shown heterogeneous alterations in alveolar epithelial developmental signaling pathways (Aubert et al. 2025), yet a unified framework for standardized interpretation of cross‐model data remains lacking. Although emerging single‐cell multiomics technologies offer the potential to resolve cellular heterogeneity (Cardenas et al. 2023), their application to CDH—particularly in samples with limited cell numbers—remains technically challenging. International consortia are needed to establish standardized experimental protocols and data standards, especially for epigenetic analyses of surrogate biospecimens such as placenta and umbilical cord blood (Chen et al. 2023).

### 8.3 Ethical Challenges: Prenatal Testing and Intervention

Advances in prenatal diagnostic technologies now enable the detection of CDH during fetal life (Didier et al. 2021; Shinar et al. 2024), raising distinct ethical considerations. On one hand, risk prediction based on epigenetic biomarkers may provoke ethical debates regarding pregnancy termination (Khalaj et al. 2022); on the other, interventions such as fetal endoscopic tracheal occlusion (FETO) require careful balancing of procedural risks against potential benefits, as evidenced by efficacy assessments in North American multicenter trials (Bergh et al. 2024). Potential epigenetic‐based interventions—such as RA supplementation—involve complex risk–benefit evaluations, given that animal models demonstrate that dysregulated RA signaling directly impairs diaphragm development (Sharma et al. 2020). An international guidance group has recommended the development of a core outcome set that includes patient representatives (Vergote et al. 2023); however, translating epigenetic discoveries into ethically sound clinical practice remains a subject requiring interdisciplinary deliberation. Moreover, the lack of safety data on epigenetic‐modifying agents during human development poses a major challenge to prenatal intervention strategies (Loukogeorgakis et al. 2025).

## 9 Translational and Clinical Prospects

From a translational perspective, epigenetic biomarkers and epigenetic‐targeted interventions are both promising yet face significant challenges. Prenatal noninvasive testing—based on maternal cell‐free DNA (cfDNA) or extracellular vesicle (EV)‐derived miRNAs—holds theoretical potential for disease stratification and prediction of severe outcomes; however, existing human studies are predominantly single‐center and underpowered, falling short of the standards required for clinical validation (Stokes et al. 2024). Meanwhile, preclinical data—such as therapeutic delivery of miR‐200b or rescue via Sin3a/HAT inhibition—provide proof‐of‐concept for developing prenatal or perinatal small‐molecule or nucleic acid–based interventions. Before advancing to clinical trials, comprehensive safety assessments in large‐animal models, long‐term follow‐up studies, and reproductive toxicology evaluations are essential (Khoshgoo et al. 2019).

Ethically and regulatorily, prenatal epigenetic interventions raise concerns regarding the risk–benefit balance between fetus and mother, the potential for transgenerational epigenetic inheritance, and the complexity of interpreting predictive information in prenatal decision‐making. Consequently, any strategy advancing to clinical application must be implemented within a rigorous ethical framework and accompanied by long‐term follow‐up plans. Short‐term feasible pathways include: (1) defining therapeutic windows and assessing safety using iPSC/organoid systems and large‐animal models; (2) concurrently establishing multicenter prenatal–postnatal cohorts to validate the predictive performance of liquid biomarkers; and (3) ultimately pursuing a phased clinical development pathway—from initial safety studies to early efficacy trials and long‐term outcome monitoring—to achieve successful translation (Fabietti et al. 2021; Kunisaki et al. 2021).

## 10 Future Directions

Current research on CDH faces significant challenges due to limited sample sizes and insufficient statistical power. To address this issue, large‐scale, multicenter EWAS are needed in the future to integrate clinical resources from diverse geographic regions. Previous studies have demonstrated that whole‐exome sequencing of 827 proband‐parent trios substantially enhances the detection rate of genetic variants (Qiao et al. 2021). Furthermore, integrating transcriptomic and metabolomic data into a multiomics analytical framework is essential. For instance, recent proteomic analyses of CDH lung tissue have identified dysregulation of the NF‐κB signaling pathway (Dylong et al. 2023), illustrating how multiomics approaches can elucidate the molecular mechanisms underlying CDH. Additionally, establishing trans‐ethnic cohorts is critically important, as epidemiological data on CDH remain scarce in regions such as China (Chen, Tao, et al. 2024b). International multicenter collaborations will be instrumental in identifying population‐specific risk factors.

Functional validation remains a critical bottleneck in CDH research (Kunisaki et al. 2021). It is therefore recommended to establish a standardized, multitiered validation framework. First, patient‐derived iPSCs should be used to model disease phenotypes; existing studies have demonstrated that CDH organoids can recapitulate abnormal lung development (Kunisaki et al. 2021). Second, such organoid platforms can be leveraged to screen for potential therapeutic targets—for example, small‐molecule interventions targeting the TGF‐β pathway (Ullrich et al. 2023). Finally, candidate mechanisms and interventions should be validated in animal models, including vitamin A deficiency models (Burns and Kardon 2023) and genetically engineered mice (Stokes et al. 2024). Notably, recent studies employing tissue‐specific knockout mice have successfully recapitulated the three hallmark features of CDH: diaphragmatic malformation, pulmonary hypoplasia, and pulmonary hypertension (Stokes et al. 2024). These gene–environment interaction models provide a powerful new tool for mechanistic investigations.

Advancing CDH research requires global data sharing (Vergote et al. 2023). We recommend establishing an international CDH registry to systematically collect standardized clinical data and biospecimens. A multicenter study involving 49 US pediatric hospitals has already highlighted the impact of racial disparities on CDH outcomes (Sferra et al. 2023), and this collaborative model should be extended to basic research. Additionally, a standardized core outcome set must be developed (Vergote et al. 2023) to enable meaningful comparisons across studies. In terms of data sharing, particular attention should be paid to epidemiological data from low‐ and middle‐income regions—especially China (Chen, Tao, et al. 2024b) and Latin America (Maia et al. 2022)—where CDH may exhibit distinct characteristics. Furthermore, we propose creating an open‐access epigenetic database that archives data from EWAS, WGBS, and related assays (Petit et al. 2023; Sharma et al. 2020), thereby accelerating the discovery of gene–environment interaction mechanisms in CDH.

## 11 Conclusion

The etiology of CDH exhibits a complex interplay between genetic susceptibility and environmental influences. Epigenetics, by mediating the regulation of gene expression, provides a crucial link for understanding this gene–environment interaction. Accumulating evidence indicates that DNA methylation, histone modifications, and noncoding RNAs all play central roles in CDH pathogenesis, intersecting with established genetic pathways—such as RA, TGF‐β, and NF‐κB signaling—to form a multilayered regulatory network.

Experimental models, including animal studies and organoid systems, are now enabling a testable causal chain: “genetic mutation → epigenetic dysregulation → developmental defect → pharmacological rescue,” marking a pivotal shift from observational associations toward mechanistic intervention. Nevertheless, the field continues to face significant challenges, including scarcity of human samples, lack of standardized data collection protocols, and insufficient evaluation of ethical and safety considerations—particularly regarding prenatal applications.

To address these gaps, future research should prioritize the following directions:

1. Large‐scale EWAS and WGBS using accessible prenatal tissues such as placenta and umbilical cord blood.
2. Integration of multiomics data coupled with cross‐model functional validation (e.g., iPSC‐derived organoids, genetically engineered animals).
3. Establishment of an international, open‐access epigenetic database and standardized research frameworks to facilitate data harmonization and reproducibility.
4. Ethically guided exploration of the safety and feasibility of prenatal epigenetic interventions under rigorous oversight.

Through these coordinated efforts, the field is poised to translate epigenetic insights into precise risk prediction and personalized therapeutic strategies for CDH, thereby advancing the clinical application of epigenetics in the study of developmental disorders.

## Author Contributions

**Yunshan Gao:** conceptualization, writing – original draft, writing – review and editing. **Xian Zhu:** literature search and screening, writing – review and editing. **Rutao Dai:** literature search and screening, writing – review and editing. **Xichen Zhang:** evidence synthesis, writing – review and editing. **Yongyu Ma:** evidence synthesis, writing – review and editing. **Ya Dao:** visualization and table/figure preparation, writing – review and editing. **Junru Chen:** writing – review and editing. **Shiwu Yang:** supervision, writing – review and editing. **Jun Wu:** supervision, project administration, writing – review and editing.

## Funding

This work was supported by the Kunming Health Science and Technology Talent Training Program – Medical Technology Center Construction Project (Project No.: 2023‐SW(Tech)‐00).

## Conflicts of Interest

The authors declare no conflicts of interest.

## Data Availability

Data sharing not applicable to this article as no datasets were generated or analysed during the current study.
